# Supplementary material for: Structural and functional insights into δ-poly-L-ornithine polymer biosynthesis from Acinetobacter baumannii
Source: Commun Biol. 2023 Sep 26;6:982. doi: 10.1038/s42003-023-05362-4 (PMC10522769; doi:10.1038/s42003-023-05362-4)
Supplement: Supplementary file 2 — Supplementary Information [file 42003_2023_5362_MOESM2_ESM.pdf]

Structural and functional insights into δ-poly-L-ornithine polymer biosynthesis from *Acinetobacter baumannii*

Ketan D. Patel and Andrew M. Gulick

Department of Structural Biology, University at Buffalo, SUNY, Buffalo, NY, 14203, United States.

Supplementary Material

Table of contents

Supplementary figures	Page
Supplementary Fig. 1: Anti-SMASH 3.0 results for PosA gene cluster showing neighboring genes and domain architecture of PosA.	4
Supplementary Fig. 2: SDS-PAGE of PosA proteins.	5
Supplementary Fig. 3: Substrate screening for PosA adenylation domain using coupled NADH consumption assay.	6
Supplementary Fig. 4: Gel filtration purification S200 16/600 profile of NRPS terminal domain with and without DDM.	7
Supplementary Fig. 5: Trans-membrane helices prediction from PosA NRPS terminal domain using CCTOP and TM-HMM programs.	8
Supplementary Fig. 6. UV chromatograms (210nm) for PosA reaction with controls.	9
Supplementary Fig. 7. EIC chromatograms overlay for 10-mer and 11-mer poly-L-ornithine.	10
Supplementary Fig. 8. EIC chromatograms for ornithine and lysine polymers from PosA reactions with L-Ornithine and L-Lysine.	11
Supplementary Fig. 9. Isocratic chromatograms for PosA δ-poly-L-ornithine product	12
Supplementary Fig. 10. Dansylation reaction to distinguish δ-poly-L-ornithine and α-poly-L-ornithine.	13
Supplementary Fig. 11. TIC chromatograms overlay for dansylation reactions.	14
Supplementary Fig. 12. EIC 252:254 overlay for Dansyl-OH from dansylation reactions.	15
Supplementary Fig. 13. MS spectra of all peaks from dansylation reaction of PosA poly-L-ornithine polymers.	16
Supplementary Fig. 14. Simulated annealing omit map electron density (green) for L-Orn (left) and D-Orn (right) contoured at 2.5 σ.	17
Supplementary Fig. 15. Superposition of PosA and εPL synthetase adenylation domain structures and substrate binding pockets.	18
Supplementary Fig. 16. Michaelis-Menten plots for PosA adenylation domain WT and mutants with L-Orn and L-Lys.	19

Supplementary Fig. 17. Comparison of substrate binding cavity between WT and model of M218A. 20

Supplementary Fig. 18. SSN of homopolymer synthetase sequences from various organisms. 21

Supplementary Fig. 19. Biofilm comparison of WT and *posA* knockout strain. 22

Supplementary Fig. 20. AlphaFold2 model of PosA C-terminal domain. 23

Supplementary Fig. 21. Sequence alignment of adenylation domains from CHPAS and Ornithine activating adenylation domain from BacB-Module 2. 24

Supplementary Tables

Supplementary Table 1. Mass of Relevant Compounds 25

Supplementary Table 2. Stachelhaus code residues comparison across different phylum from adenylation domains of CHPAs 26

Supplementary Table 3. Sequence of PosA from A. baumannii 27

Supplementary Table 4. Primers used in the study 28

Supplementary Table 5. Codon optimized gene fragments synthesized in the study 29

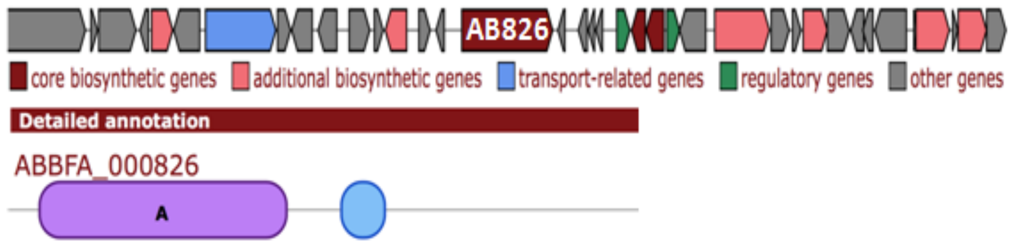

Supplementary Fig. 1. Anti-SMASH 3.0 results for PosA gene cluster showing neighboring genes and domain architecture of PosA. *Acinetobacter baumannii* strain AB307-0294 complete genome sequence, GenBank: CP001172.1 (replaced by CP001172) was submitted to Anti-SMASH. PosA (originally annotated as ABBFA_000826) was the only core NRPS biosynthetic gene in the cluster. PosA domain architecture showed presence of adenylation domain (purple), thiolation domain (sky blue) and terminal domain could not be predicted.

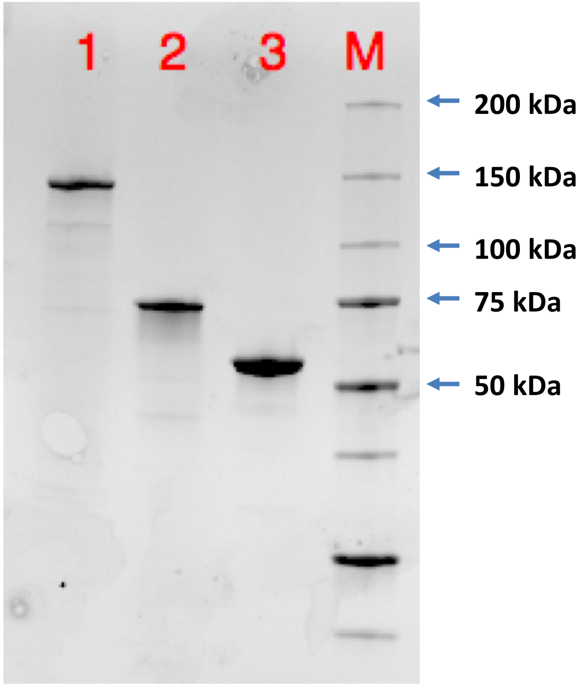

Supplementary Fig. 2. SDS-PAGE of PosA proteins. 1: PosA-full length protein (1-1332, 148 kDa), 2: PosA truncated terminal domain (604-1332 residues, 80.8 kDa), 3: PosA truncated adenylation domain (1-512 residues, 57.0 kDa). Purified proteins were loaded over SDS-PAGE.

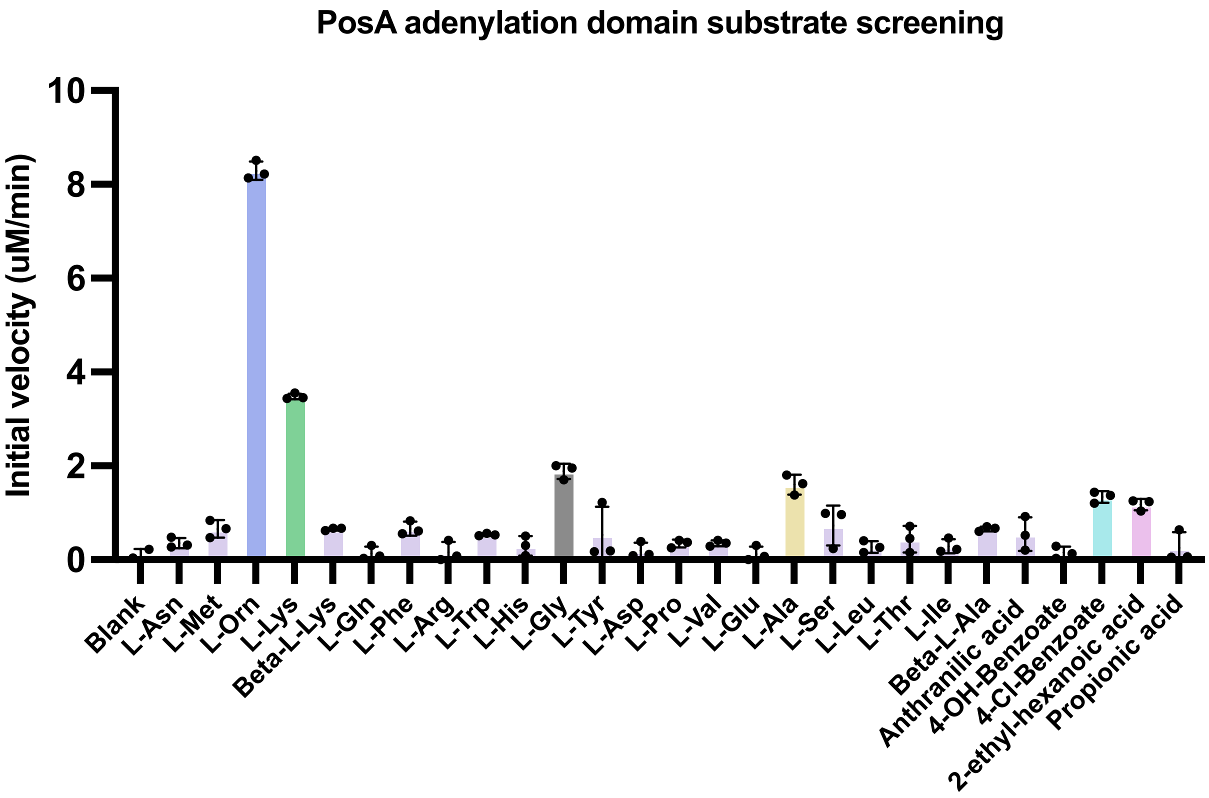

Supplementary Fig. 3. Substrate screening for PosA adenylation domain using coupled NADH consumption assay. Amino acids, fatty acids, and non-proteogenic amino acids were tested at 5 mM with 1 μM PosA adenylation domain to observe coupled NADH consumption. L-Ornithine (L-Orn) showed highest NADH consumption followed by L-Lysine (L-Lys). Standard error bars are presented for triplicate measurements.

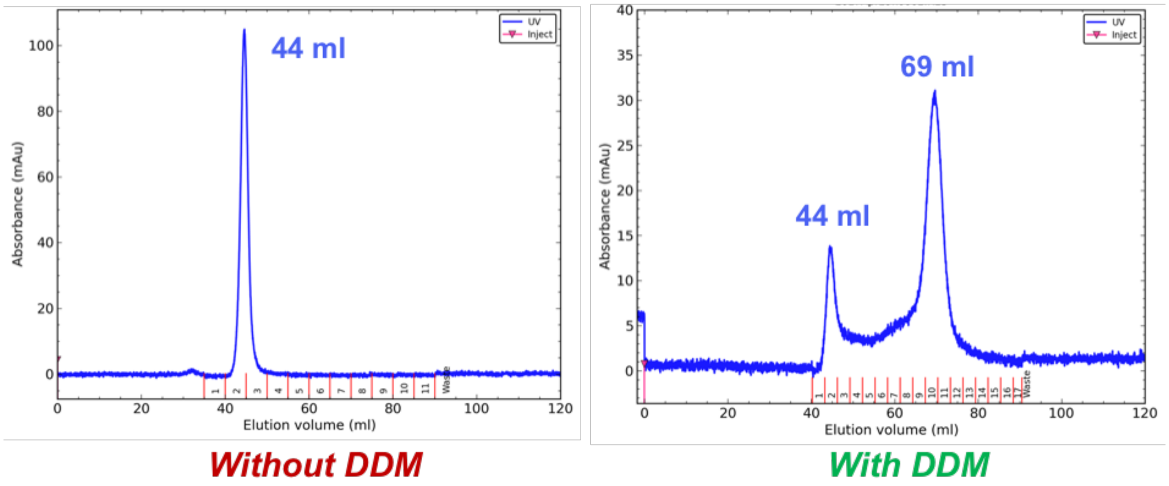

Supplementary Fig. 4. Gel filtration purification S200 XK 16/600 profile of NRPS terminal domain with and without DDM. Isolated NRPS terminal domain when purified as soluble domain without any detergent, all protein elutes as aggregation peak at 44 ml in gel filtration with Superdex S200 XK16/60 column after IMAC purification (left panel). However, addition of 1% DDM detergent during lysis and 0.25% in purification buffers results in another peak at 69 ml in gel filtration corresponding to 155 kDa molecular weight matching to dimeric 84.2 kDa of isolated PosA NRPS terminal domain (right panel).

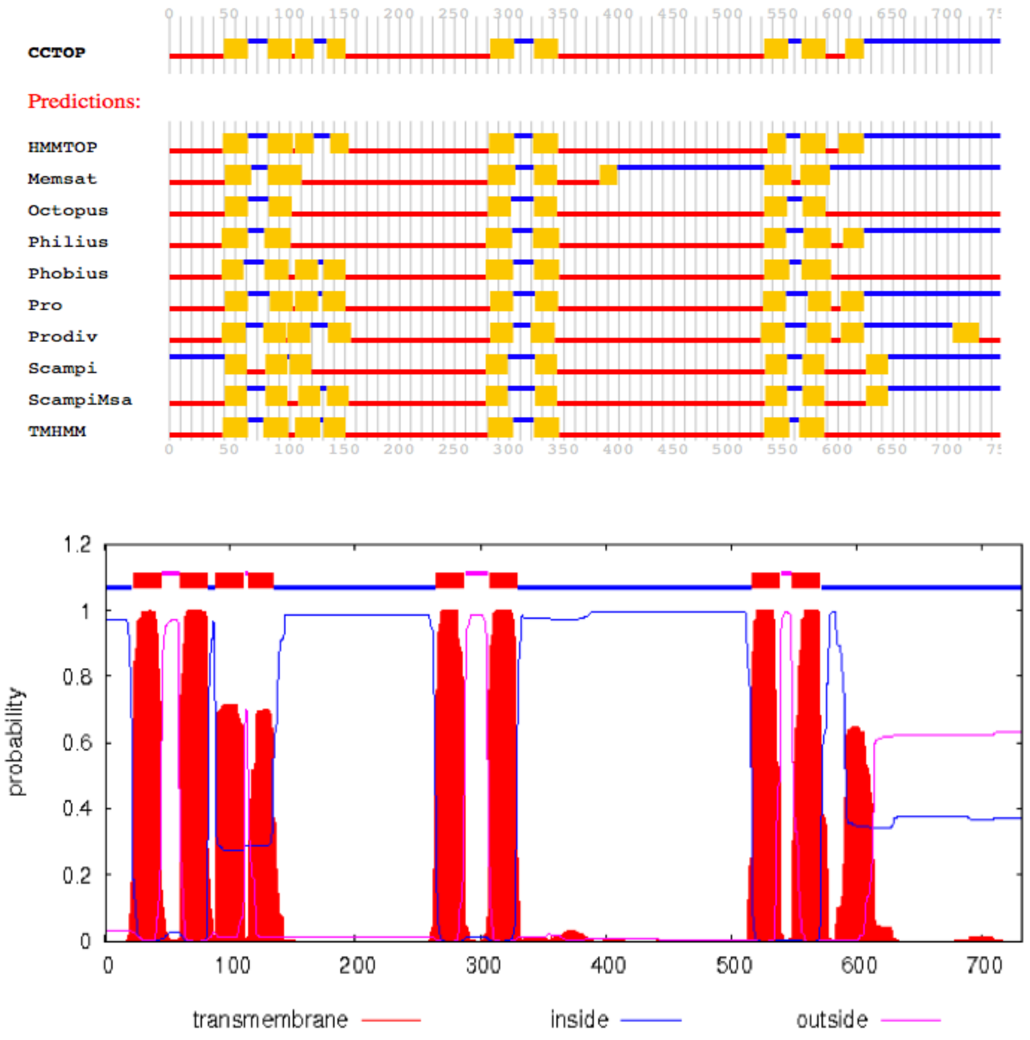

Supplementary Fig. 5. Trans-membrane helices prediction from PosA NRPS terminal domain using CCTOP (top panel) and TM-HMM (bottom panel) programs. To observe presence of trans-membrane helices in PosA NRPS-terminal domain, sequence analysis was carried out using online programs CCTOP, top panel, and TM-HMM, bottom panel. PosA protein sequence (ATY43264.1) from 604 to 1332 was submitted to each program. The yellow boxes in top panel and red boxes in lower panel indicate propensity trans-membrane helix in the protein sequence. Six high-confidence trans-membrane helices were predicted in the PosA NRPS-terminal domain.

CCTOP https://cctop.ttk.hu
TM-HMM https://services.healthtech.dtu.dk/services/TMHMM-2.0/

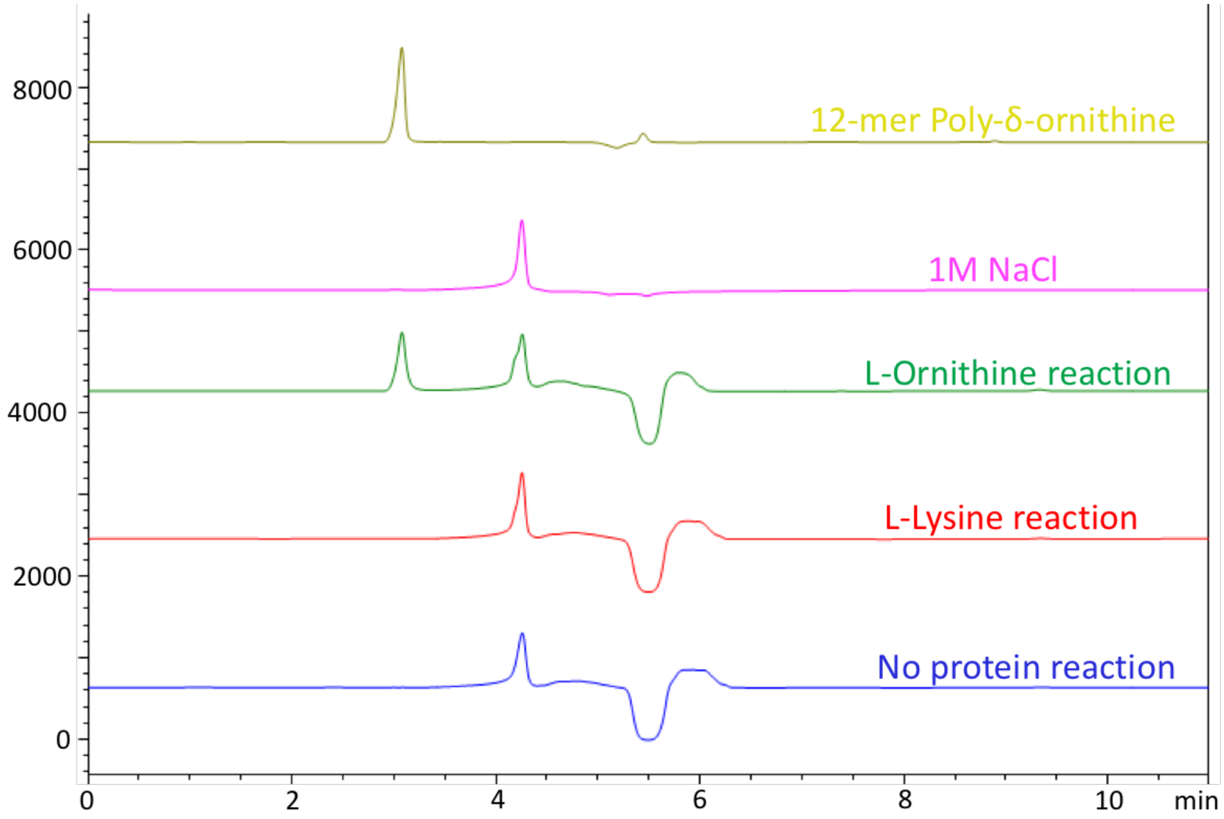

Supplementary Fig. 6. UV chromatograms (210nm) for PosA reaction with controls. To identify the poly-L-ornithine peak from PosA reaction, 12-mer δ-poly-L-ornithine (12-δPO) standard was used. Corresponding 2.0 ml fractions from SP cation exchange chromatography 1M NaCl wash of L-Ornithine reaction, L-lysine reaction, and protein-free control reactions were dried as described in materials and methods and subjected to LC/MS analysis. L-ornithine reaction with PosA (green) showed two peaks at 3.0 ml and 4.2 ml. 12-δPO standard (gold) showed 3.0 ml peak indicating the corresponding peak in L-ornithine reaction as poly-L-ornithine. However, the 4.2 ml peak present in L-ornithine reaction, L-lysine reaction (red) and protein-free control reaction (blue) matched to the peak in 1M NaCl standard.

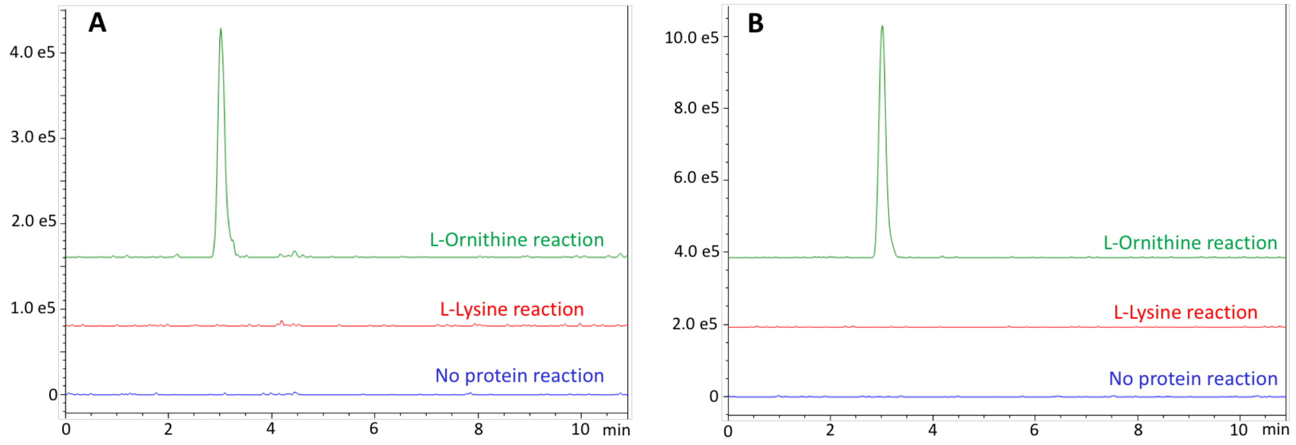

Supplementary Fig. 7. EIC chromatograms overlay for 10-mer and 11-mer poly-L-ornithine. A) Extracted Ion Chromatograms (1159:1161) representing the 10-mer of poly-L-ornithine and B) 1272:1274 representing the 11-mer of poly-L-ornithine. EIC peaks for 1160 and 1273 m/z average were observed in L-ornithine reaction samples (green), but not in L-lysine reaction (red) or protein-free control reaction (blue).

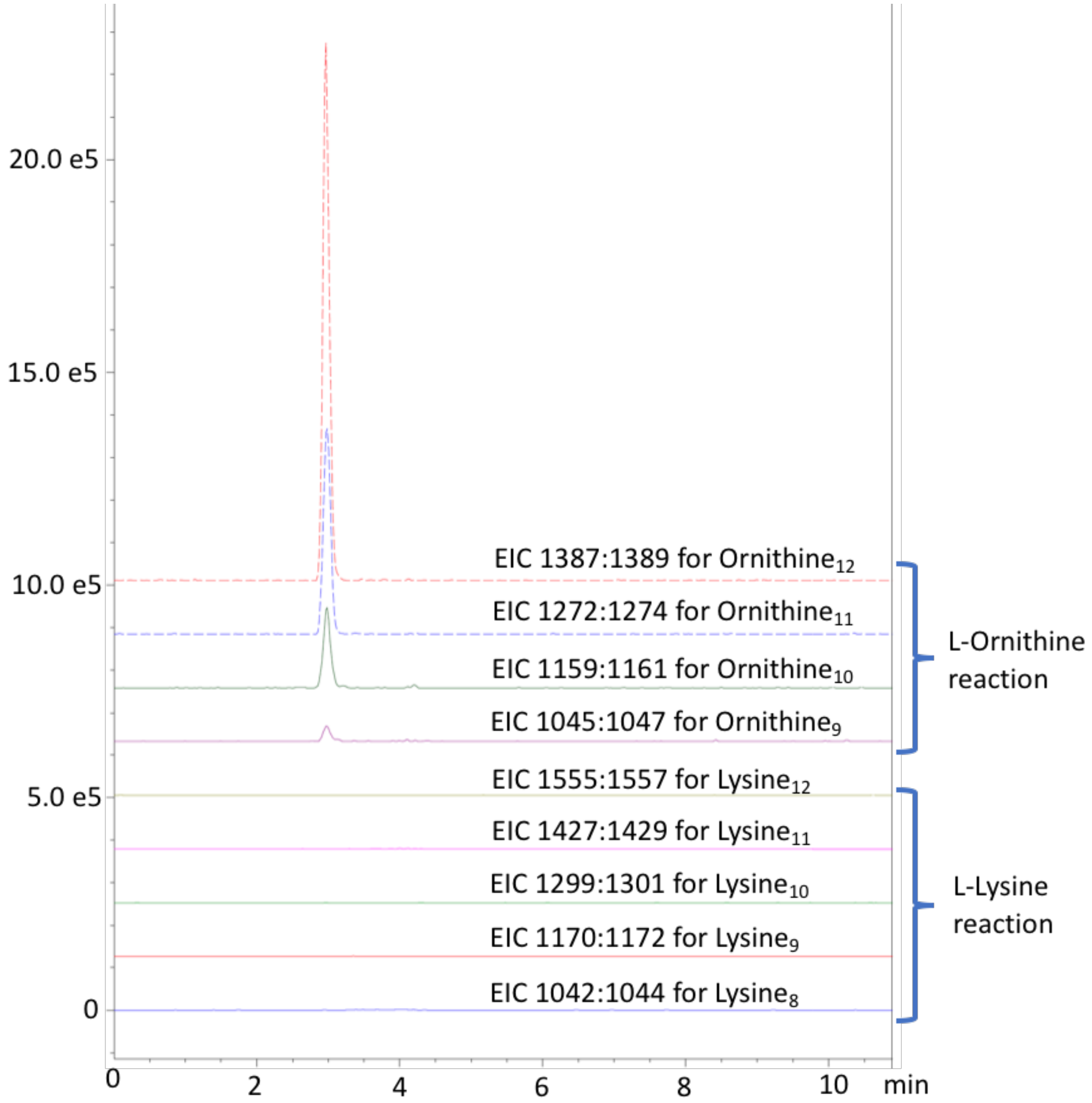

Supplementary Fig. 8. EIC chromatograms for ornithine and lysine polymers from PosA reactions with L-ornithine and L-lysine respectively. EIC chromatograms from PosA reactions with L-ornithine showed peaks for 1046, 1160, 1273 and 1388 m/z averages representing 9-mer, 10-mer, 11-mer and 12-mer respectively. EIC chromatograms from PosA reactions with L-lysine did not show peaks for 1043, 1171, 1300, 1428 and 1556 m/z averages representing 8-mer, 9-mer, 10-mer, 11-mer and 12-mer respectively.

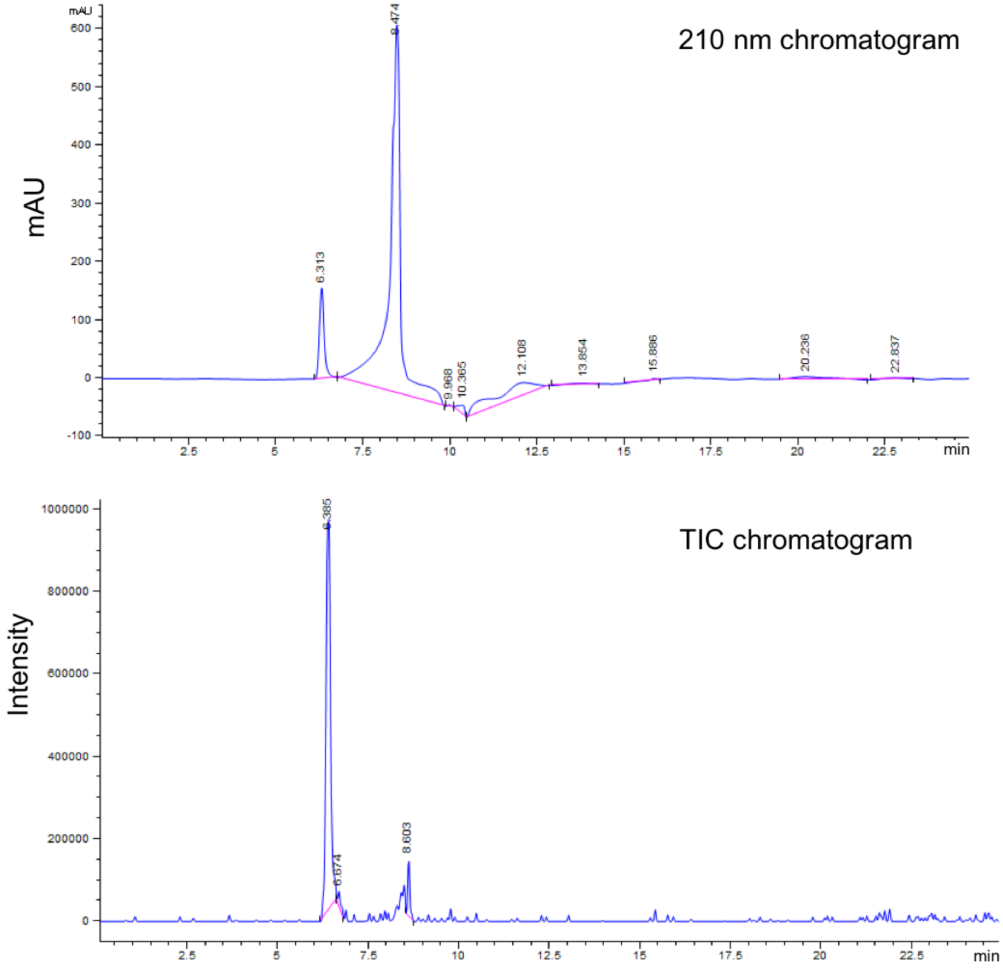

Supplementary Fig. 9. Isocratic chromatograms for PosA δ-poly-L-ornithine product. An optimized isocratic separation and increased sample volume was used to obtain the MS spectrum shown in Fig. 2C. UV absorbance, 210 nm (top panel) and Total Ion Current chromatograms (bottom panel) for PosA concentrated product. PosA enzymatic reaction product was purified over SP cation exchange chromatogram and concentrated by drying as described in materials and methods. Concentrated product was loaded onto Poroshell 120-EC C18 reverse-phase 4.6x100mm column. HPLC method was isocratic run of 50% acetonitrile in water with 0.1 % formic acid for 25 min at 0.25 ml/min. Peak at 6.3-6.4 min showed MS spectra for 7-mer to 12-mer of δ-poly-L-ornithine as shown in figure 2C. The absorbance peak at 8.5 min was present in samples consisting only of the 1M NaCl as well.

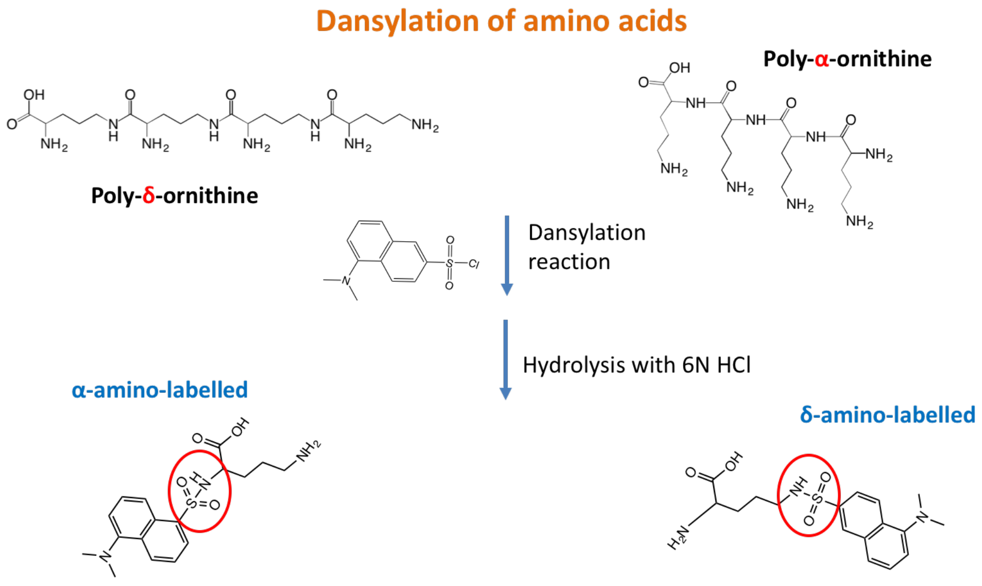

Supplementary Fig. 10. Dansylation reaction to distinguish δ-poly-L-ornithine and α-poly-L-ornithine. 12-mer δ-poly-L-ornithine standard and 12-mer α-poly-L-ornithine standard were used in dansylation reactions to distinguish α-amino peptide-bonding and δ-amino iso-peptide bonding in poly-L-ornithine polymers from PosA enzymatic reaction. Mixing the polymers with dansyl-Cl and incubation makes covalent bond between free amines and dansyl group. Subsequent treatment with 6N HCl hydrolyses the peptide bond but not covalent bond of dansyl-amine. This results in release of the monomeric N-dansyl-L-ornithine. Depending on the availability of free amine groups in the polymer, the δ-poly-L-ornithine yields α-amino labeled N-dansyl-L-ornithine, while the α-poly-L-ornithine yields δ-amino labeled product. α- and δ-labeled N-dansyl-L-ornithine elute at different times in HPLC runs allowing the differentiation between two types of bonding in poly-L-ornithine.

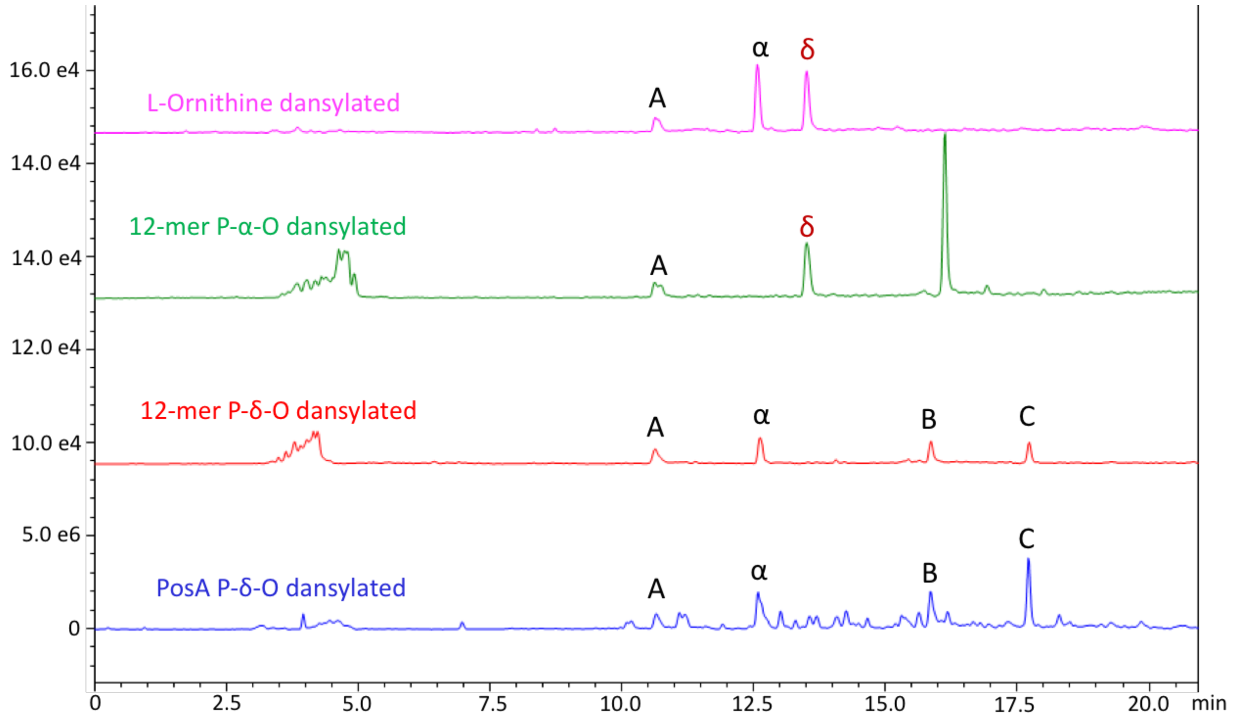

Supplementary Fig. 11. TIC chromatograms overlay for dansylation reactions. Dansylation reactions were performed, followed by acid hydrolysis, and TIC chromatograms were overlaid for L-ornithine (magenta), 12-mer α-poly-L-ornithine standard (green), 12-mer δ-poly-L-ornithine standard (red), and the poly-L-ornithine product of the PosA reaction (blue). Other peaks were also observed at 10.6, 15.8 and 17.7 mins labeled as A, B and C. Peaks at 12.6 and 13.5 represent α-*N*-dansylornithine and δ-*N*-dansyl ornithine, respectively. Peak A, B and C represent the dansyl-OH, dimeric amino labeled N-dansyl-L-ornithine and trimeric amino labeled N-dansyl-L-ornithine, see Supplementary Fig. 13.

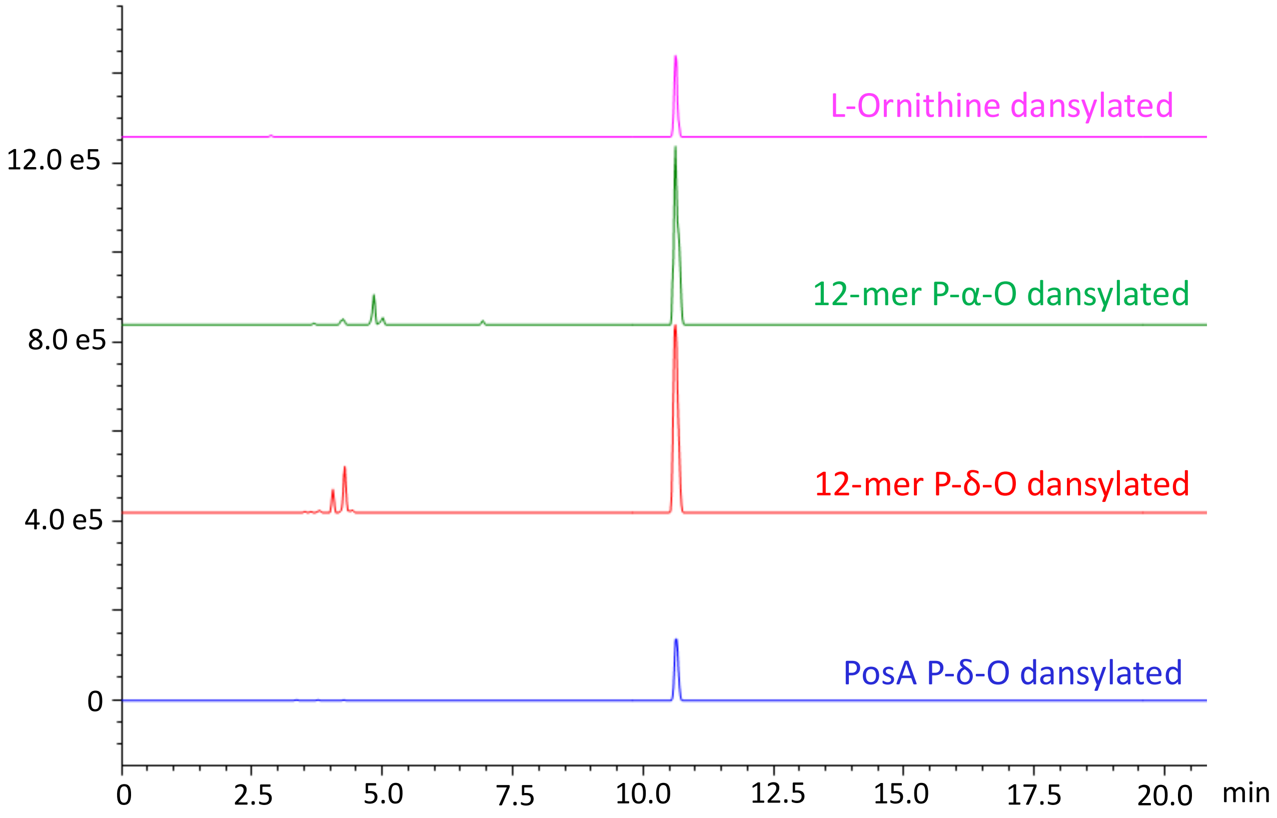

Supplementary Fig. 12. EIC 252:254 overlay for Dansyl-OH from dansylation reactions. EIC for 252:254 showed peak at 10.6 min in all samples indicating formation of by-product dansyl-OH in all reactions. Dansylation reactions for L-ornithine (magenta), 12-mer α-poly-L-ornithine standard (green), 12-mer δ-poly-L-ornithine standard (red) and PosA poly-L-ornithine (blue) are shown.

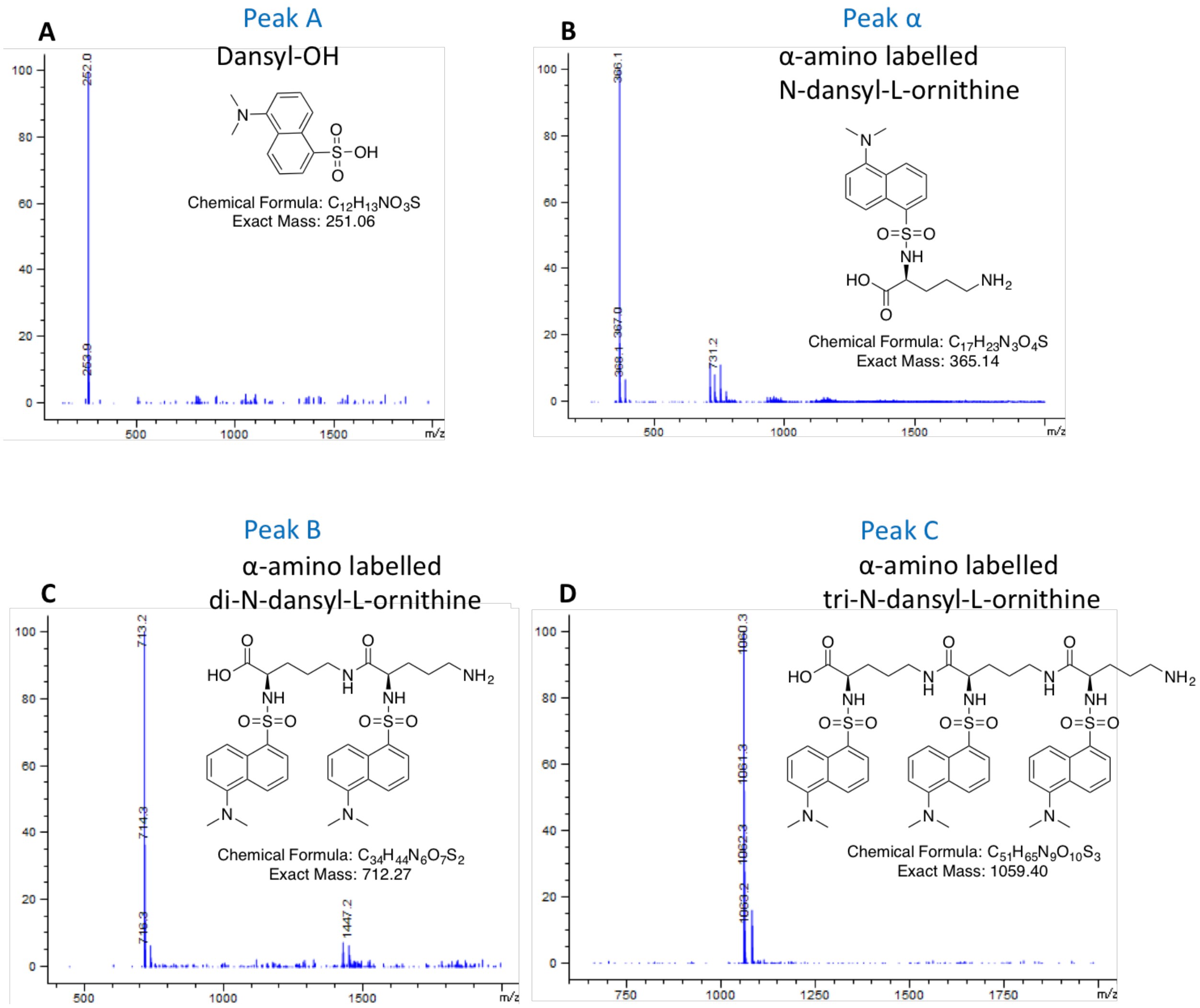

Supplementary Fig. 13. MS spectra of all peaks from dansylation reaction of PosA poly-L-ornithine polymers. A) Peak A MS spectra 252.0 m/z representing the dansyl-OH. B) Peak α MS spectra of 366.1 m/z representing the α-amino labeled N-dansyl-L-ornithine. C) Peak B MS spectra 713.2 m/z representing α-amino labeled di-N-dansyl-L-ornithine. D) Peak C MS spectra 1060.3 m/z representing α-amino labeled tri-N-dansyl-L-ornithine.

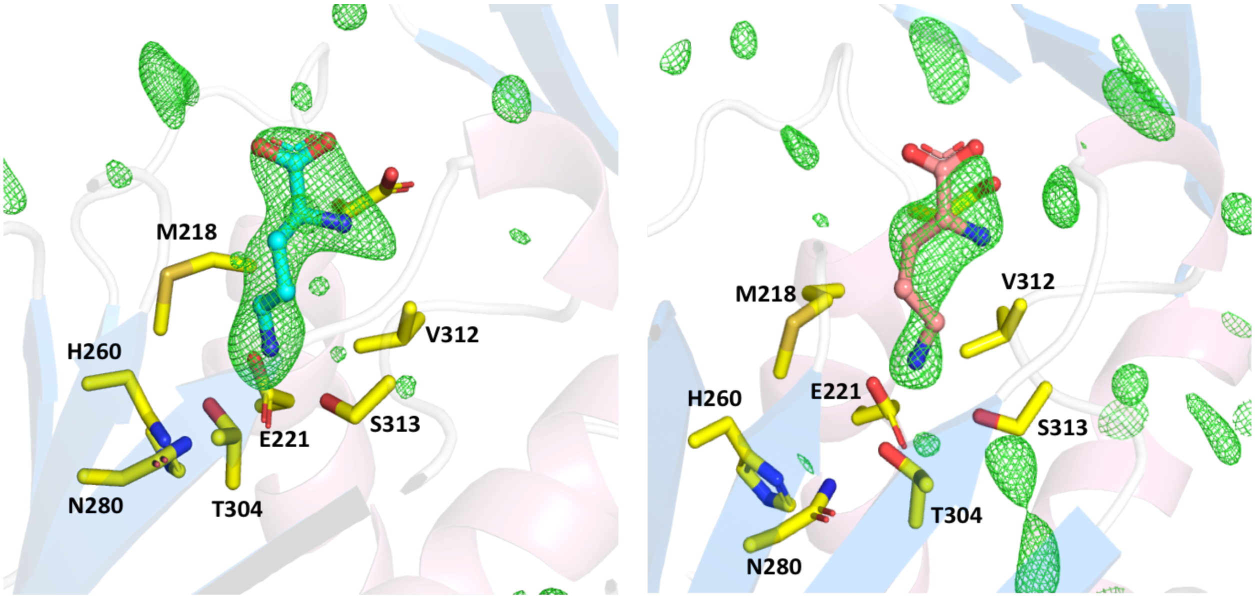

Supplementary Fig. 14. Simulated annealing omit map electron density (green) for L-Orn (left) and D-Orn (right) contoured at 2.5 σ.

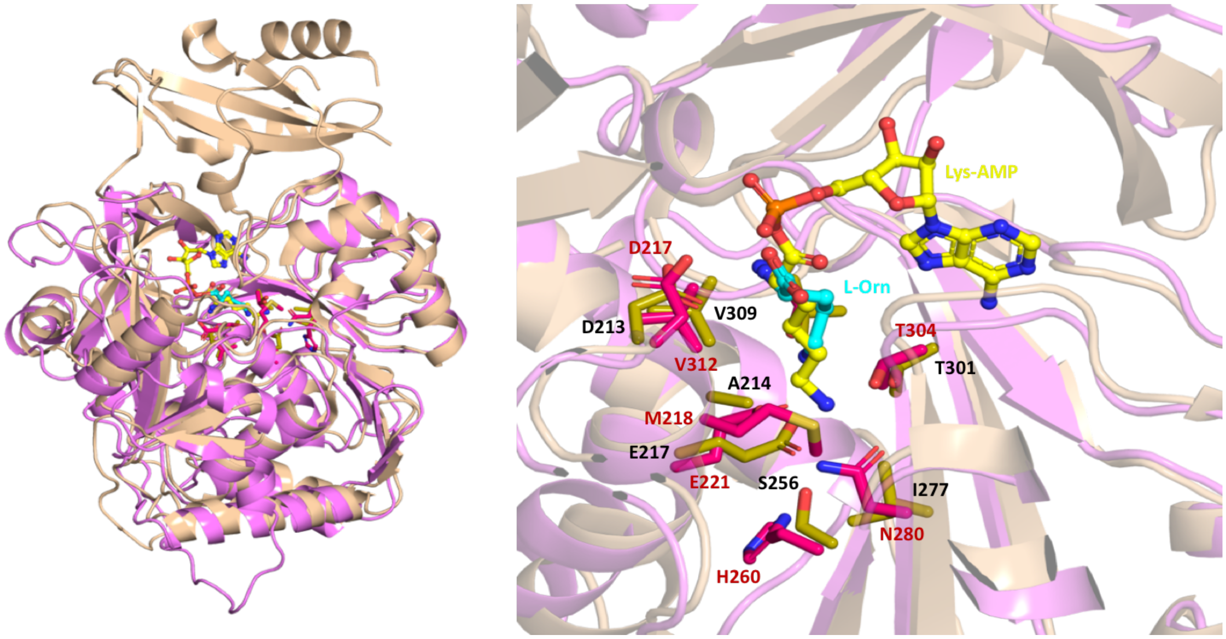

Supplementary Fig. 15. Superposition of PosA and εPL synthetase adenylation domain (PDB 7WEW) structures and substrate binding pockets (right). Left panel shows full length adenylation domain structures from εPL synthetase (wheat) and N-terminal domain of PosA adenylation domain (pink) superposed. Right panel shows substrate binding residues from adenylation domains of εPL synthetase (pink carbon) and PosA (wheat carbon) along with substrates Lys-AMP (yellow carbon) and L-orn (cyan carbon) respectively.

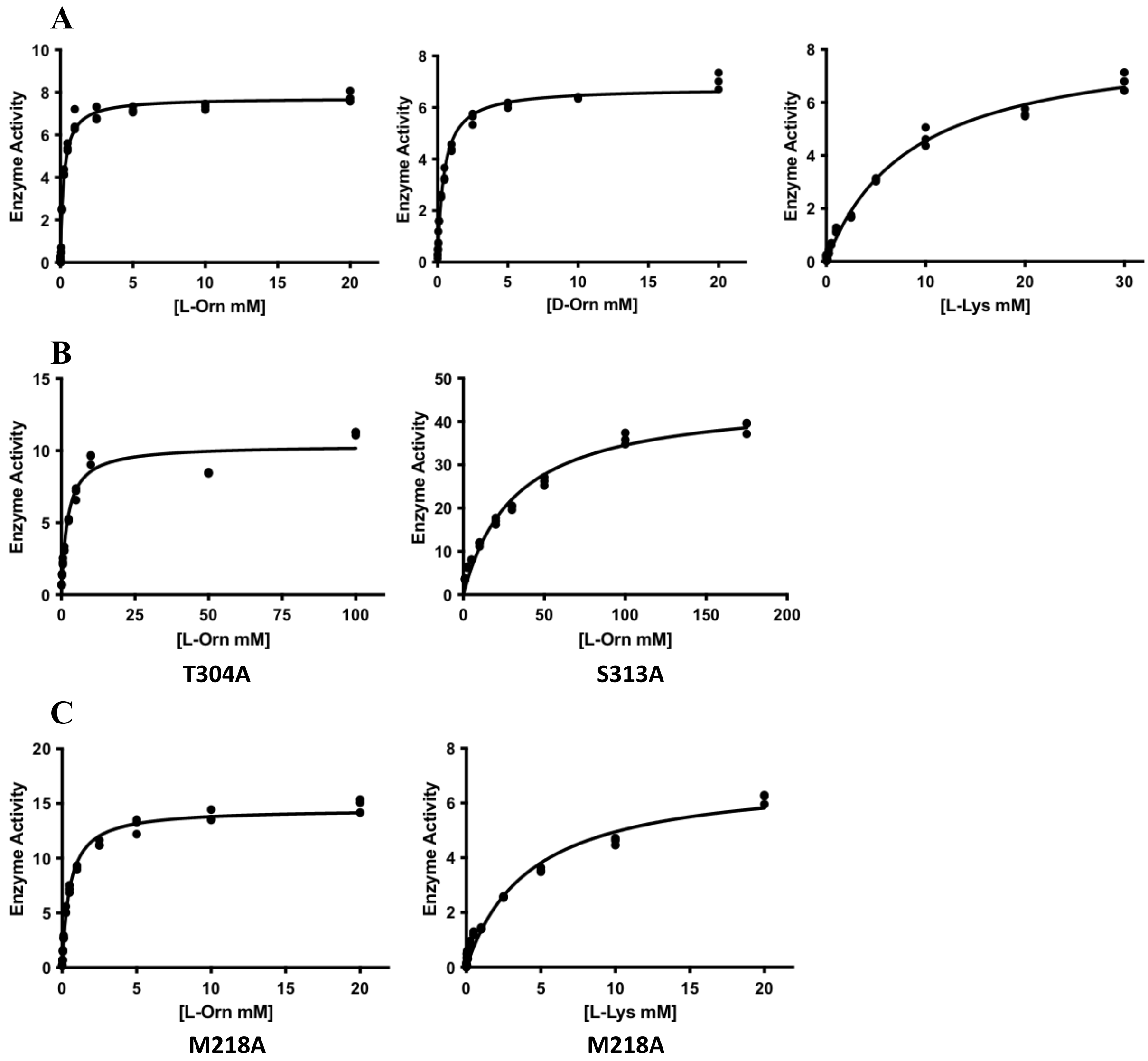

Supplementary Fig. 16. Michaelis-Menten plots for PosA adenylation domain WT and mutants with L-ornithine (L-Orn), D-ornithine (D-Orn) and L-lysine (L-Lys). A) PosA WT showed sufficient activity for L-Orn and D-Orn, while lower activity with L-Lys. B) Since mutants did not showed saturation at 1 μM protein concentration, a higher concentration was used for T304A and S313A to obtain apparent kinetic constants. C) M218A showed higher activity with L-Orn and improved activity with L-Lys as described in Table 1. Triplicate data points are shown at each substrate concentration.

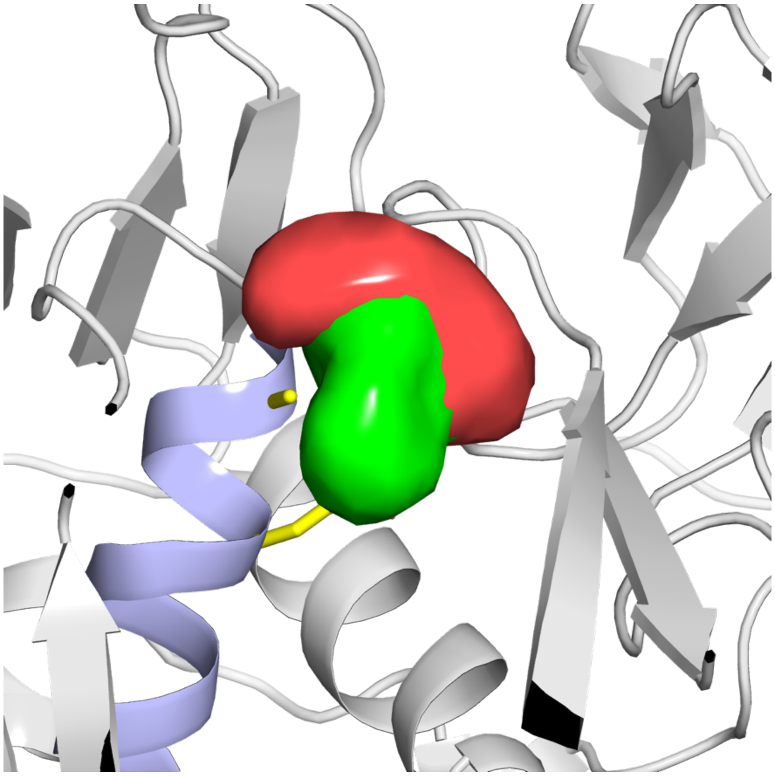

Supplementary Fig. 17. Comparison of substrate binding cavity between WT and model of M218A. Structure of wild type was used to truncate M218 side chain to Ala in PyMol. Cavities for WT and M218A structures were generated by program Hollow using cylindrical constraint mode (PMID: 19014592). Substrate binding cavity in WT (colored red) and modelled Ala218 residue (yellow) in mutant (colored green) shows extra volume created by truncation of Methionine to Alanine. The extra volume (green color) may provide room for one extra carbon in L-lysine compared to L-ornithine which may lead to improved binding and catalysis of L-lysine in M218A mutant compared to WT.

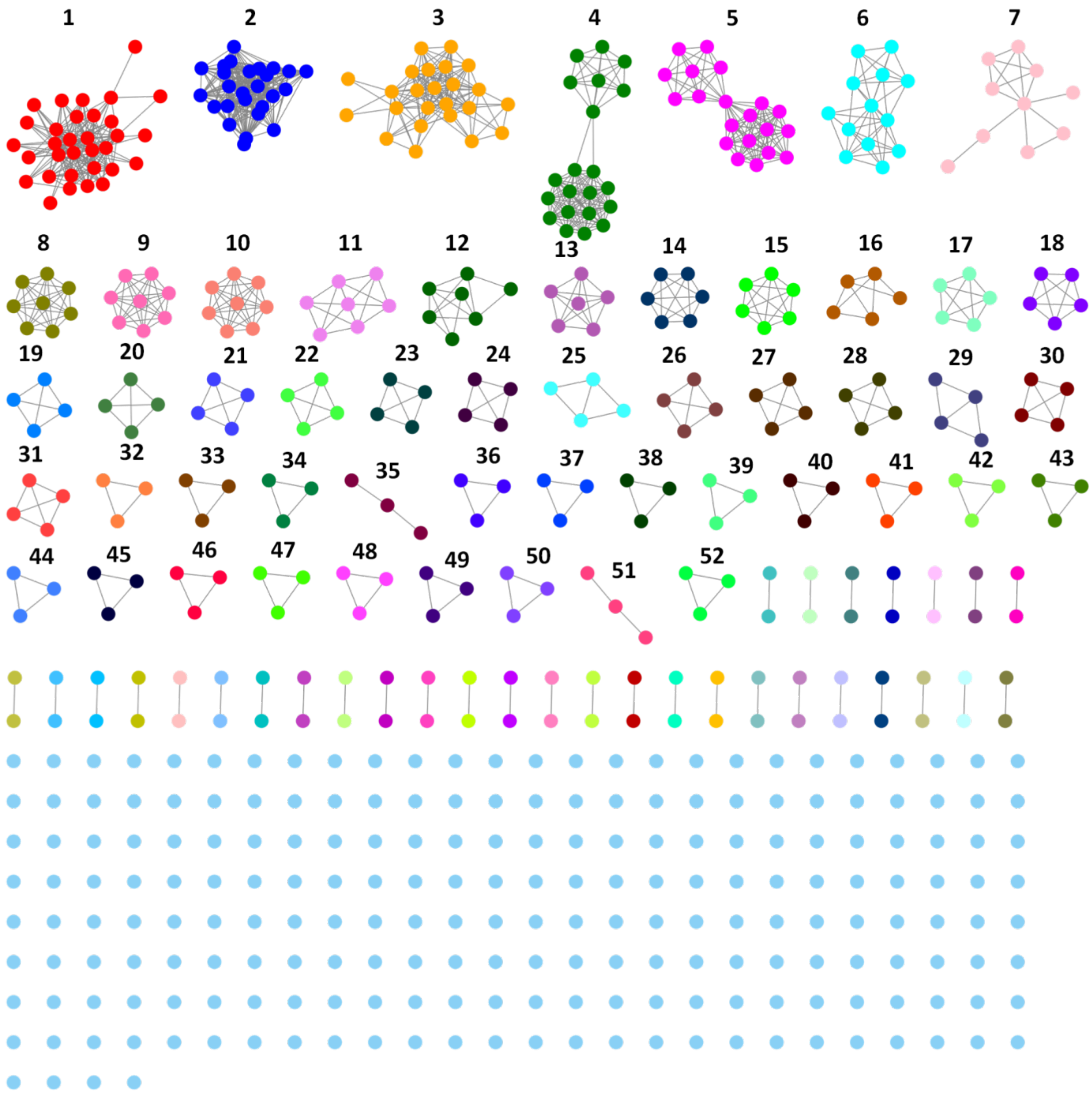

Supplementary Fig. 18. SSN of homopolymer synthetase sequences from various organisms. 536 sequences of bacterial and eukaryotic phyla collected from BLAST and HMMER searches were used to generate SSN network. Alignment score threshold of 525 was used to create clusters. PKS NRPS sequence analysis (20) from each cluster was performed to find out Stachelhaus code residues to predict probable substrate and hence the homopolymer (Supplementary Table 2). Sequences from cluster 1, 6 & 7 representing Streptomyces and other Actinobacteria showed Stachelhaus code residues matching to εPLs, while those from cluster 2 representing alpha-proteobacteria matched to PDAP synthetase. Cluster 3 & 5 sequences representing beta & gamma proteobacteria harbored the same Stachelhaus active site sesquence as poly-L-ornithine synthetase from *A. baumannii*. Interestingly, fungal clusters 4 & 9 showed residues matching to εPLs.

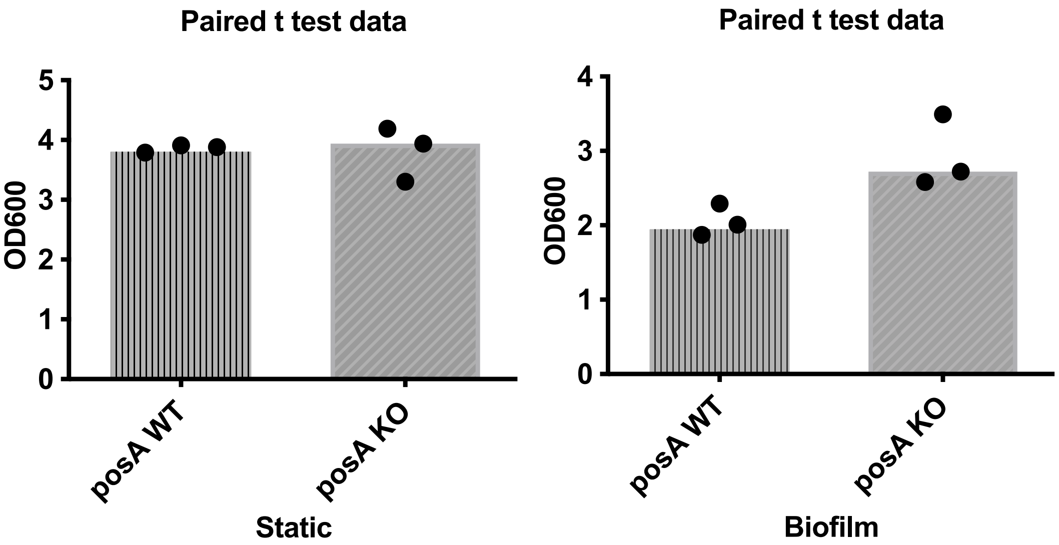

Supplementary Fig. 19. Biofilm comparison of WT and *posA* knockout strain in L.B. medium. OD_{600} measurements from planktonic cells grown without shaking were considered for static growth and cells bound to the bottom surface were considered biofilm. Biofilm measurements were performed as described in materials and methods. Statistical significance difference between wild type (WT) and *posA* gene knockout (KO) was determined using paired t-test. Significant difference ($p<0.05$) was observed in biofilm formation of *posA* gene knockout strain relative to WT strain (right panel) but not in static culture (left panel).

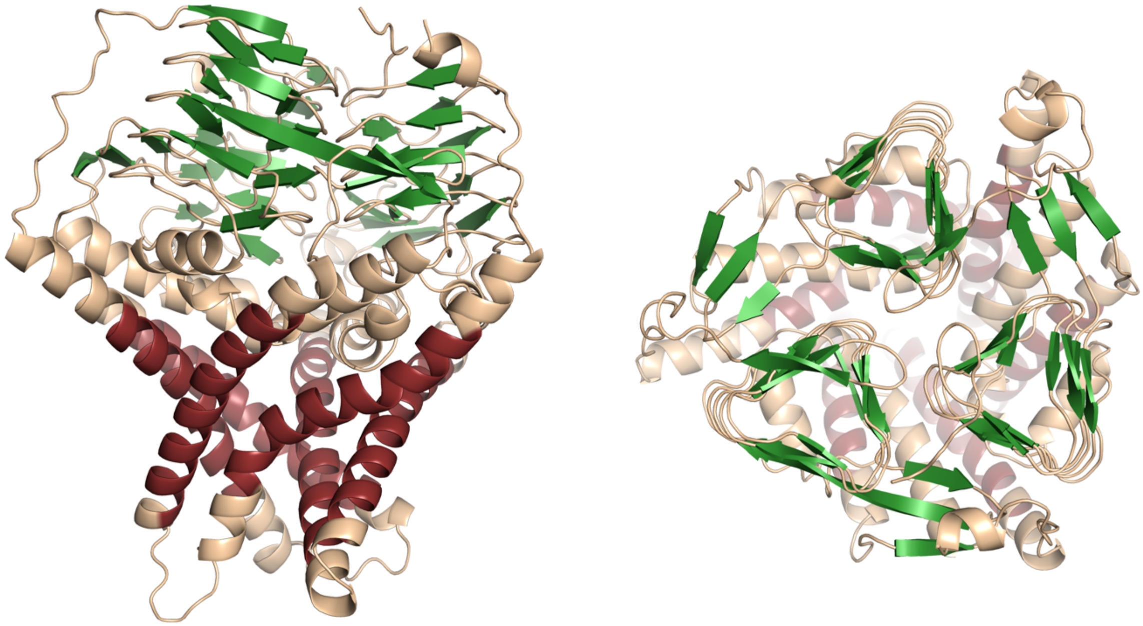

Supplementary Fig. 20: AlphaFold2 model of PosA C-terminal domain. Regions predicted to be TM helices are colored deep red. Two orthogonal views are shown.

```
                                           217-218 221
εPL_synthetase      LFCQDQPLGP-GDRVLAGLSVAFDASCEEMWLAWRYGACLVPAPRAL-VRAGHELGPWLV 248
BacB-M2_A           HYE----LASFSVNLLQLASMSFDVFAGDLCRSLLNGGTMYIVPDDV-KLEMNLLYDMIN 253
δPO_synthetase      ILG----IQE-QDKVYQGFSVAFDMSFEEIWLSYLVGATLWIAPKSL-VSDPERLCQTLK 252
εPBL_synthetase     VCG----TGQ-GDRVYQGFALSFDGSVEEIWMAFSNGSTLVVPGRDA-PRFGEELGRYLT 241
PDAP_synthetase     VYD----VRP-DDRVYQGMTLSFDFSIEEIWPTWAVGATLVAGPVGAGARLGAELAGVLE 265
PDAB_synthetase     IYQ----VAE-PDRVYQGLSIAFDFSIEEIWPAWIAGATLIAGPADD-RRVGQGLTEFLI 263
PDDAB_synthetase    IYG----VTA-RDRVYQGMSISFDFHLEEIWPAWVAGATLVAGPTDA-RRFGQALADFLN 262
                                 .:    :::**    ::  :   *. :              *   :

                           260                   280 283
εPL_synthetase      ERGITVVSTVPTLAALWPD------EAMRRVRLLIVGGESCPAG----LVDRFAGPGREM 298
BacB-M2_A           KYGIHMLESTPSLIIPLMKYIDHHKLDFSSMKLLIMGSDTCTIKDYKWLVERFG-QRMRI 312
δPO_synthetase      QEQITVLHAVPTLLALFPE-------DVPNLRIINLGGEMCPDS----LVDRWALPHHQM 301
εPBL_synthetase     ELGITYFSTVPTLLATLPQ-------DVPSIHTLVLSGEACPPE----LVNRWARPGRRL 290
PDAP_synthetase     ASGITLFYCVPTLLATIPR-----DWELPAVRSIVVGGEPCPAR----LVERWSRPGRRM 316
PDAB_synthetase     EHAVTVLCCVPTLLTTIEA-------ELPVLRSLLVSGEACPPD----LVRRWDRPGRRI 312
PDDAB_synthetase    ENQITVFCAVPTLLTTIES-------EPDSVHTLLVSGEAMPPD----LVRRWWRPDRRI 311
                       :  .  .*:*                  :: : :..:         ** *:      .:

                     304    312-313
εPL_synthetase      WNTYGPTETTVVACAARLL-----PGEPVRIGLPLKGWQLAVVDRTGQPVPFGAEGELLI 353
BacB-M2_A           INSYGVTEASVDSGYYEEALDRIPEIANTPIGKPLDNTAFYILDPSLNPQPVGVYGELYI 372
δPO_synthetase      FNTYGPTETTVSASLELLE-----RGKPVTIGKPLPNYGMLVINSERELLEQGETGELCI 356
εPBL_synthetase     LNVYGPTEATVNTTAAECL-----PGEPVTIGRPLPGYHVTIVDEDLRPVPTGTSGELLI 345
PDAP_synthetase     LNTYGPTETTVTATVAELR-----PGRPVTIGRPVPTYTAVLLDDRREPVPDGAVGEICV 371
PDAB_synthetase     LNAYGPTETTVTATCGELL-----ANRDVTIGRPLPTYRVYLLDEELRPVPEGASGEICV 367
PDDAB_synthetase    LNCYGPTEATVSASCCELT-----PERPVTLGTPFPTYVFYVLDEDFRQVPEGETGEICI 366
                     * ** **::* :               . :* *.      :::   .    *  **: :
```

Supplementary Fig. 21. Sequence alignment of adenylation domains from CHPAS and Ornithine activating adenylation domain from BacB-Module 2. Numbers on top indicates residue numbers in PosA adenylation domain. Boxes over the residues shows conservation of Stachelhaus code residues. The terminal amine interacting glutamate residue at Stachelhaus code position 3 (residue 221 in PosA) is conserved in all CHPAs. However, in BacB-Module 2 adenylation domain position 3 is replaced with glycine and glutamate is observed at position 4 (residue 260 in PosA).

Supplementary tables

Supplementary Table 1. Mass of Relevant Compounds

Compound	Chemical Formula (protonated form)	Calculated $(M+H)^+$	Observed $(M+H)^+$
**Diamine Polymers**			
Ornithine	$C_5H_{13}N_2O_2^+$	133.1	--
$Ornithine_7$	$C_{35}H_{73}N_{14}O_8^+$	817.5	817.5
$Ornithine_8$	$C_{40}H_{83}N_{16}O_9^+$	931.7	931.6
$Ornithine_9$	$C_{45}H_{93}N_{18}O_{10}^+$	1045.7	1045.7
$Ornithine_{10}$	$C_{50}H_{103}N_{20}O_{11}^+$	1159.8	1159.8
$Ornithine_{11}$	$C_{55}H_{113}N_{22}O_{12}^+$	1273.9	1273.8
$Ornithine_{12}$	$C_{60}H_{123}N_{24}O_{13}^+$	1388.0	1388.8
Lysine	$C_6H_{15}N_2O_2^+$	147.1	--
$Lysine_8$	$C_{48}H_{99}N_{16}O_9^+$	1043.8	--
$Lysine_9$	$C_{54}H_{111}N_{18}O_{10}^+$	1171.9	--
$Lysine_{10}$	$C_{60}H_{123}N_{20}O_{11}^+$	1300.0	--
$Lysine_{11}$	$C_{66}H_{135}N_{22}O_{12}^+$	1428.0	--
$Lysine_{12}$	$C_{72}H_{147}N_{24}O_{13}^+$	1556.2	--
**Dansyl-labeled Ornithine**			
N-Dansyl-L-ornithine	$C_{17}H_{24}N_3O_4S^+$	366.2	366.1
Dansylhydroxide	$C_{12}H_{14}NO_3S^+$	252.0	252.0
N,N-didansyl-L,L-diornithine	$C_{34}H_{45}N_6O_7S_2^+$	713.3	713.2
N,N,N-tridansyl-L,L,L-triornithine	$C_{51}H_{66}N_9O_{10}S_3^+$	1060.4	1060.3

-- Not detected

Supplementary Table 2. Stachelhaus code residues comparison across different phylum from adenylation domains of CHPAs

Organism	**Domain/phylum**	**Stachelhaus code**	**Substrate**
Acinetobacter baumannii (ATY43264.1)	γ-Proteobacteria	D M E H N G T V S K	**Orn***
Streptomyces albulus (pls) (BAG68864.1)	Actinobacteria	D A E S I G T V V K	**Lys***
Streptomyces celluloflavus (BCD58482.1)	Actinobacteria	D F E C L S A V T K	**DAB***
Streptomyces albulus PD-1 (EXU85975.1)	Actinobacteria	D F E Y V G T V T K	**DAP***
Streptoalloteichus hindustanus (WP_234995776.1)	Actinobacteria	D G E S V S V V N K	**β-Lys***
Streptoalloteichus hindustanus (WP_083959783.1)	Actinobacteria	D F E C L S C V S K	**D-DAB***
Pseudomonas syringae (MCF9016611.1)	γ-Proteobacteria	D M E H N G T V S K	Orn
Oxalobacteraceae bacterium (NRR32876.1)	β-Proteobacteria	D M E H N G T V S K	Orn
Rhizobium tropici (MBN8953344.1)	α-Proteobacteria	D F E A L G T V V K	DAP
Pedobacter caeni (WP_073234317.1)	Acidobacteria	D M E H N G S V T K	Orn
Acidobacteria bacterium (PYV28785.1)	Acidobacteria	D A E S I G T V I K	Lys
Streptomyces cavourensis (RBL79953.1)	Terrabacteria	D M E H N G T V S K	Orn
Mycobacterium tuberculosis (PLV54557.1)	Terrabacteria	D A E S I G T V V K	Lys
Chitinophaga vietnamensis (WP_201747873.1)	Bacteroidetes	D M E H N G S V S K	Orn
Fortiea contorta (WP_069108566.1)	Cyanobacteria	D A E S I G T V V K	Lys
Scytonema tolypothrichoides (WP_048867422.1)	Cyanobacteria	D F E Y I G T V T K	DAP
Embleya scabrispora (WP_235619347.1)	Actinobacteria	D F E C L S A V T K	DAB
Aspergillus arachidicola (PIG69232.1)	Fungi	D A E S I G T V V K	Lys
Dothistroma septosporum (EME39875.1)	Fungi	D M E N N G T V T K	Orn
Piloderma croceum (KIM77850.1)	Fungi	D M E H I L T V S K	Orn
Planoprotostelium fungivorum (PRP79759.1)	Amoebozoa	D A E S I G S V V K	Lys
Nematostella vectensis (XP_001625520.1)	Metazoa	D A E S I G S V V K	Lys
Vitrella brassicaformis (CEM14083.1)	Alveota	D A E S I G T V V K	Lys
Podila minutissima (KAF9322632.1)	Actinobacteria	D F E G I G T I T K	DAP
Mobilicoccus pelagius (WP_217994546.1)	Actinobacteria	D F E L M G T V T K	DAP
Ktedonobacteraceae bacterium (MBO0777672.1)	Terrabacteria	D F E C L G T V T K	DAP
Singulisphaera sp. (WP_254053915.1)	Planctomycetes	D F E F V G T V T K	DAP
Sulfuriferula plumbiphila (WP_147073502.1)	β-Proteobacteria	D F E C N G T A S K	#
Mesorhizobium plurifarium (CDX48809.1)	α-Proteobacteria	D F E A L G T V T K	DAP
Citricoccus sp. Ch26a (WP_010143560.1)	Actinobacteria	D G E S V S V V N K	#
Methylomarinum sp. (NOR71114.1)	γ-Proteobacteria	D G E S I S V V N K	#§
Novosphingobium sp. (WP_072382105.1)	α-Proteobacteria	D S E C Y G G V T K	#
Bdellovibrionales bacterium (OFZ39577.1)	γ-Proteobacteria	D S E C Y G G V T K	#§
Methylobacterium sp. (WP_056379773.1)	α-Proteobacteria	D L E D L G T V T K	#
Rhizobium hainanense (WP_075855952.1)	α-Proteobacteria	D L E D I G T V V K	#
Thaumarchaeota archaeon (OLE40053.1)	Archaea	D L E S I G T V I K	#
Botryobasidium botryosum (KDQ08654.1)	Fungi	D I E S I G T V T K	#

Letters in bold green indicates conserved residue for di-amino substrate activation. Letters in bold red indicates variable residues important for substrate specificity. *, experimentally confirmed substrates. (Orn: L-ornithine, Lys: L-lysine, DAP: diamino propionate, DAB: diamino butyrate, D-DAB: D-diamino butyrate). #, uncharacterized binding pocket. §, targets chosen for biochemical analysis.

Supplementary Table 3. Sequence of PosA from *A. baumannii* AB307-0294

NCBI Locus ATY43264 (1332 amino acids)

```
   1   MNQFVTNTKN VIRGKYHPEF LQNEVLADIF AHTAQTLPDK TALIEADKTL SYGELYQQAL
  61   IMAQHLALKG VKPGHIVGLW LPRGIELLKA QLAICLSGAA WLPFDMDTPA DRIAVCLEDA
 121   EAVGMITTDE WYEHLAEVPQ TKWTNTELQK PLSESVSLAK TTPDQPAYII YTSGSTGKPK
 181   GIVITQKNIC HFLRSENSIL GIQEQDKVYQ GFSVAFDMSF EEIWLSYLVG ATLWIAPKSL
 241   VSDPERLCQT LKQEQITVLH AVPTLLALFP EDVPNLRIIN LGGEMCPDSL VDRWALPHHQ
 301   MFNTYGPTET TVSASLELLE RGKPVTIGKP LPNYGMLVIN SERELLEQGE TGELCIFGPS
 361   VAQGYLGRPD LTADKFIENP WAMSVEEELL YRTGDLAKID EFGQVHCLGR ADDQVKIRGF
 421   RVELGEIEAA LCDIDGIGTA AVILRPEDGI DQLIAFIAPE IDAKQAIEIK ELRHNLSQRL
 481   PPYMVPNRFE IIEEVPRLLS GKIDRKALKA RPLTSVVDRS ESDQPQNPAE EILFEILNRL
 541   FPNMPIKLDS DFFDDLGGHS LLAAVLISNL REHAEYSHLT IQNLYQARRV GAIAALMLEQ
 601   PEPTLFDSQI GQDNPRNQTY KWLCGIAQLV TIPVLISINI LQWLAPFFTY HYFTGGTRDS
 661   IPYAIALSLL VYVSVIMSSF VLSITVKRLL MLGIGAGRYP LWGLTYFRWW LADRISNISP
 721   VYLLSGSTLL NLYLKALGAK IGHDVTISSV HIRMPSLLTI EDGVSIGSQV NLENAKVEHG
 781   HLVLGSIHLK QDSYVGSYAV LEENTVLEKQ AHVNALTSIE YDTVVPEGEI WDGTPAQKIG
 841   HIDEQAKLPE RPKLSFIRKI AEYGYYGVSA LIIACLFFIP IFPSFLLVDW LDVNVFNINP
 901   NNHLQIALYY FILAIPASAM MMITAVISS GLRKIALPRL EIGTYAVHGS TYYRKWFAAQ
 961   ILETSLQTLH GLFATIYAPT WFRMLGAKVG KNTEISTATG VIPEMLTLGE ESFIADAVML
1021   GDEEIKGGWM SLKATKIGNR SFVGNSAYIA DGTVLPDNVL IGVQSKTPDN REMYDGQTWF
1081   GSPALLLPAR EAAEKYPDHL TFKPSIKRRL MRGFIEGLRI VLPAALAIGV GYMIVLDVID
1141   VINNYNIETG LVALTLAGLL YGVGCFLIVA LLKWILIGRY QPRSAPMWTM FVWLSEGITS
1201   LYESVAIPNF LNYLRGTPML PFFLRILGVR IGKDVYMDTA DITEFDCVSI GDRAEFNSFS
1261   GPQTHLFEDR IMKIGQVNVG NDVVVNTRSI ILYNANVSNH AVLGPLTLVM KGENIPAKSA
1321   WIGSPAVPWV HK
```

Three domains of PosA illustrating adenylation domain (blue), carrier domain (black, with pantetheinylation motif highlighted, and C-terminal transmembrane domain (green).

Supplementary Table 4. Primers used in the study

Primer name	Sequence 5′-3′
PosA_Full_F	TTTCAGGGCCATATGAACCAGTTTGTAACGAACACG
PosA _Full_R	AGCCGGATCCTCGAGTTATTTATGAACCCAAGG
PosA _A_R	GCCGGATCCTCGAGTTACGGTCTTGCTTTTAAAGC
PosA _DUF_F	TTTCAGGGCCATATGACTTTATTTGATAGCCAA
AB_A_D217A_F	CGCTTTTGCGATGTCGTTTGAAGAGATTTGGCTTTC
AB_A_D217A_R	CTTCAAACGACATCGCAAAAGCGACTGAAAAGCCTTGATAGACC
AB_A_E221A_F	GTCGTTTGCGGAGATTTGGCTTTCTTATTTGGTAGGTGC
AB_A_E221A_R	GAAAGCCAAATCTCCGCAAACGACATATCAAAAGCGACTGAAAAGCC
AB_A_S313A_F	CACGGTTGCCGCAAGTCTTGAGTTATTAGAACGAGGC
AB_A_S313A_R	CTCAAGACTTGCGGCAACCGTGGTTTCGGTTGGTCCATAGG
AB_A_T304A_F	GTTTAACGCCTATGGACCAACCGAAACCACGG
AB_A_T304A_R	GGTTGGTCCATAGGCGTTAAACATTTGATGATGAGG
AB_A_M218A_F	CGCTTTTGATGCGTCGTTTGAAGAGATTTGGCTTTC
AB_A_M218A_R	CTCTTCAAACGACGCATCAAAAGCGACTGAAAAGCCTTG
AB_Kan_F	CATTTAGCTGAAGTGCCGCAAACAAAATGGACCAACACCGAGCTGCAAAAACCATTAA GCGAAAGTGTAAGTTTAGCGAAAACCACTCCAGATCAACCTGCCTATATTATTTATAC CTCGGGTTCGAAAGCCACGTTGTGTCTCAAAATC
AB_Kan_R	GATTACGAGGGTTATCTTGTCCAATTTGGCTATCAAATAAAGTAGGTTCAGGCTGCTC TAACATGAGTGCAGCAATCGCGCCTACTCTTCTCGCTTGATATAAGTTTTGAATGGTC AGATGGCTATACATTATTCCCTCCAGGTATTAGA
AB_KO_seqF	CCGAAGCAGTAGGAATGATTAC
AB_KO_seqR	CATAATGGATAACGCCCTGC
Bdello_A_F	TTTCAGGGCCATATGAACCAGAAC
Bdello_A_R	AGCCGGATCCTCGAGTCAGTCGAC
Methylo_A_F	TTTCAGGGCCATATGAATAGTATCTC
Methylo_A_R	AGCCGGATCCTCGAGTCAGGGAGT

Supplementary Table 5. Codon optimized gene fragments synthesized in the study

Genes	Sequence
Bdellovibrionales bacterium	**TTTCAGGGCCATATG**AACCAGAACTTATTGCTGCACCAGATCTTTGAAGCAACGGCCAAG<br>AAGTATCCACAACAGATTGCACTTGATATCCCCCCGCTGAGTACACGCCCTCGCATCTGT<br>CTGACTTATAAAGAGTTAGACCGTATGGCTAACTACCTTGCTGGTCAGATTGCACCCTTT<br>GTAAAGGCGAATTCTGAAACAGTAATCGCGCTTCTGTTAGAACGTAACAGTCACCACCTT<br>TTCGTCGCTCAATTGGCTGCGCTGAAAGCTGGGGCAGCCTACTGCAGCATTGACTCGTCT<br>TTCCCCGCGGAACGCATCTCATTTATTTTAGATGACTCCAAAACACCGCTGATTCTTACA<br>ACAGAGAGTCTTCGCCAGCTTCTTCCTAATGGATACCATACCATTGACGTCGCAAATGCT<br>TTAACTAACATCGCCAACGACAAAACTTATGACTTCGTAACGCCCACATGGGTTCTTGCT<br>AATAATCTGGCCTACATCATTTACACATCGGGTACTACAGGCAAACCGAAGGGAGTCGCC<br>TGCGAGCATCGCAATATCGTAAATTTAATTCAGGCCGACGTAGACTACTTCAAGTTAAGT<br>AGTAGCGAACGCATCTCACAGAATTCCAGTACGTCTTACGACTCTTCATTAGAGGAGATC<br>TGGATGGCCTGGGCTATTGGGGCTACGGTCGTTGTGGCCACAGACGAAATTGTACGTTCA<br>GGACCGGACTTCGGGGCATGGTTAAAAAAAGAGAAAATCACTGTCATCTGTCCACCACCT<br>ACTCTGTTAAAAATGAAAGTTGCAACAAACCCGAAAGTAGATTTACCAGACTTGAAGTTA<br>GTTTATGTAGGGGGGGAAGCCTTGCCTCGTGAGATCGCCGATTTGTGGGGGCGTGACCTT<br>TGGCTTGAAAATGGTTATGGCCCTACCGAATGCACAGTAACCGTGACCCGCACGCGCATC<br>TTCCCACAGCAGGCTATTAGCATCGGACGCCCCGTAAAAAATAACTTCGCGTTAATCCTT<br>GATGAGAAGTTAAACGAGTTGAAAATTGGAGAGATTGGTGAGCTGTGTATTGGTGGAGCT<br>CAGTTGACTCGCGGCTATCTTCATCGCCCTGAACTGACAAATGAAAAGTTTATTACTCAT<br>CCACTGCATGGCCGCATTTACCGCACAGGAGATTTGGCAAGTAAGAATGCCGATGATGAG<br>ATCTTCTACCACGGACGTGCAGACTCACAGGTTAAACTTCGCGGTTATCGCATCGAACTT<br>GGGGACATTGAGTCAGCAATTTCGACATGTGAAAATGTGGCTGCAGCGGCGTGCAAGGTG<br>CAAGAGTCTCGTGCGGGACAACGCTTAATCGCGTACTTAGTGTCCAAGGATAACTGCGCG<br>GTTGATCTTGAGAAGATCAAATCCACTCTGGAGAAGAACCTTCCGAAATATATGATTCCA<br>AGTTTATTCATGCAATTGTCGAAACTTCCCTTGTTGGCGACTTCTGGTAAGCTGGACCGC<br>AAGGCTTTGCCGGACCCTGAGGAAAATACAAATTTGGTCGACTGA**CTCGAGGATCCGGCT**
Methylomarinum sp	**TTTCAGGGCCATATG**AATAGTATCTCACCAGTGCTTGCCCCCGAAACGAAGGCAGAGTGT<br>CTGCACCATCTTTTTGAGGCACAAGTAATCATTCGCCCCACCCATTTGGCTGTCATCTAT<br>GGAGAGGAACGTTTGTCCTATCAAGAGTTGGACTACCGTGCTAATCAATTGGCGCAGTAC<br>CTGCGTCGTTTGGGTGTGAAACCTAATACATTGGTGGGGATTTATTTAGATCGTTCGGAA<br>AAACCAATCATTGCGATTTTAGCTATTTTGAAGGCTGGAGCTGGCTACGTACCCTTAGAC<br>CCCGATTATCCCCAGGATCGCATTGAGTACATTATCAAGGAGGCAGGAATTTCTGTTTTA<br>GTCAGCGAAGAAAAGTTGTACTCCAAAGTCTCAAGTTTTTTCGATGGGATTACAGTAACT<br>CTGGACTCGCAATGGGGCGGGATCGGTCAGCAGAGCAAAGATCTGATCTTTAGCAAGTCA<br>ACAGAAAGCAGCAGCAATCAGTTATGCTACGTTCTGTACACTAGCGGAACTACGGGGCGT<br>CCAAAGGGAGTAATGACGGAACATCATAACGTAGTTCAGTTCGTAGCTTCCTTTAATGAG<br>ATCTGTAAAATCGAACCGACCGACCGTATTTATCAAGGCTTTTCTCTTGGTTTTGACGGA<br>AGTGTAGAGGAGATCTGGATGGCCTTCTCGAATGGAGCAACCCTTATTGTTGGCCCCCCC<br>GATATTAGTCGCTTAGGGAACGAGGTAGCGGATTTCCTGACACAGCACGAGGTCACTTTT<br>TTTTCCACCGTCCCAACTTTACTGGGCATGATTTCAGAAGACTTACCGACGGTCCGTCTT<br>CTGATTGTTTCTGGAGAGCCTTGTCCCCCGCAGCTTGTTAAGAAGTGGGCCCAACCTCAA<br>CGTCGTATGCTGAATGTGTACGGCCCTACCGAGACCACAGTCAACACAACGGTTGCTGAG<br>TGCATCGCGGATAAAACTATCACGATTGGCCAACCTCTGCGCGGTTATCAAACGTTTATC<br>CTGGATTCGAATATGAAACTTTTGCCCTCGGGGGAGTCCGGAGAACTTTACATCGGGGGC<br>GTGGGTGTGGCGCGCGGCTACCTTAACCAACCGGAAATTACGGCACGCCATTTTTTGTCA<br>AATCCGTTCGACTCATCCAGTTTGTCTAAATTATACCGTACAGGTGACCTTGTACGTATG<br>AAAGATGGTGGGGACTTGGAGTTCCTTGGGCGCATGGATTCACAAGTAAAGATTCGTGGT<br>TACCGCATTGAGTTGTCAGAGATCGAGTCCGTTTTGAAAGAACATAGTGACGTACACTCC<br>GCCGTAGTCAATGTATTCGAGAACAACGGACTGAAGCAGTTAACTTCCTACGTCGTGCCT<br>TCTGTGAGTCAGGCACTTTTTGACCGCGATGATGTATTAAAGCTGTTGCGTATGCGTCTG<br>CCACCATATATGGTACCAGGTTACCTTGATTTGTTAGACGAACTGCCTACGTTGGCCAGC<br>GGCAAAGTCGACCGCAAACAGCTGCCTGAGCCTAAGACTCCCTGA**CTCGAGGATCCGGCT**

Sequence in bold represents cloning sites. Synthesized gene fragments were cloned into a pET15b-TEV plasmid at the NdeI/XhoI restriction sites to include an *N*-His-tag with TEV protease cleavage site in the expressed protein.

Protein	Primary Protein Sequences of N-His5-expressed proteins
Bdellovibrionales bacterium	MGSSHHHHHSSGENLYFQGHMNQNLLLHQIFEATAKKYPQQIALDIPPLSTRPRICLTYK<br>ELDRMANYLAGQIAPFVKANSETVIALLLERNSHHLFVAQLAALKAGAAYCSIDSSFPAE<br>RISFILDDSKTPLILTTESLRQLLPNGYHTIDVANALTNIANDKTYDFVTPTWVLANNLA<br>YIIYTSGTTGKPKGVACEHRNIVNLIQADVDYFKLSSSERISQNSSTSYDSSLEEIWMAW<br>AIGATVVVATDEIVRSGPDFGAWLKKEKITVICPPPTLLKMKVATNPKVDLPDLKLVYVG<br>GEALPREIADLWGRDLWLENGYGPTECTVTVTRTRIFPQQAISIGRPVKNNFALILDEKL<br>NELKIGEIGELCIGGAQLTRGYLHRPELTNEKFITHPLHGRIYRTGDLASKNADDEIFYH<br>GRADSQVKLRGYRIELGDIESAISTCENVAAAACKVQESRAGQRLIAYLVSKDNCAVDLE<br>KIKSTLEKNLPKYMIPSLFMQLSKLPLLATSGKLDRKALPDPEENTNLVD
Methylomarinum sp	MGSSHHHHHSSGENLYFQGHMNSISPVLAPETKAECLHHLFEAQVIIRPTHLAVIYGEER<br>LSYQELDYRANQLAQYLRRLGVKPNTLVGIYLDRSEKPIIAILAILKAGAGYVPLDPDYP<br>QDRIEYIIKEAGISVLVSEEKLYSKVSSFFDGITVTLDSQWGGIGQQSKDLIFSKSTESS<br>SNQLCYVLYTSGTTGRPKGVMTEHHNVVQFVASFNEICKIEPTDRIYQGFSLGFDGSVEE<br>IWMAFSNGATLIVGPPDISRLGNEVADFLTQHEVTFFSTVPTLLGMISEDLPTVRLLIVS<br>GEPCPPQLVKKWAQPQRRMLNVYGPTETTVNTTVAECIADKTITIGQPLRGYQTFILDSN<br>MKLLPSGESGELYIGGVGVARGYLNQPEITARHFLSNPFDSSSLSKLYRTGDLVRMKDGG<br>DLEFLGRMDSQVKIRGYRIELSEIESVLKEHSDVHSAVVNVFENNGLKQLTSYVVPSVSQ<br>ALFDRDDVLKLLRMRLPPYMVPGYLDLLDELPTLASGKVDRKQLPEPKTP

Underlined sequence represents N-5x-His-tag and TEV cleavage site residues (ENLYFQ^G) contributed by vector.
