# Supplementary material for: Structural and functional insights into δ-poly-L-ornithine polymer biosynthesis from Acinetobacter baumannii
Source: Commun Biol. 2023 Sep 26;6:982. doi: 10.1038/s42003-023-05362-4 (PMC10522769; doi:10.1038/s42003-023-05362-4)
Supplement: Supplementary file 3 — Description of Additional Supplementary Files [file 42003_2023_5362_MOESM3_ESM.pdf]

Description of Additional Supplementary Files

File name: Supplementary Data 1
Description: Source data behind the graphs in Figures 1C, 1D, and Supplementary figures 3, 16, and 19.
